# Lower Urinary Tract Symptoms in Women with Surgically Confirmed Endometriosis: Association with Urethral Ultrasonographic Findings

**DOI:** 10.3390/jcm15145603

**Published:** 2026-07-17

**Authors:** Katarzyna Tomczyk, Małgorzata Kampioni, Magdalena Adamczyk, Katarzyna Klimaszyk, Małgorzata Kędzia

**Affiliations:** Department of Reproduction and Gynecology, Poznan University of Medical Sciences, 61-701 Poznan, Poland

**Keywords:** endometriosis, pelvic floor ultrasonography, lower urinary tract symptoms

## Abstract

**Background:** Endometriosis is a chronic inflammatory disease affecting approximately 10% of women of reproductive age and is commonly associated with pelvic pain and infertility. Increasing evidence indicates that lower urinary tract symptoms (LUTS) are more common in women with endometriosis, even in the absence of direct urinary tract involvement; however, the underlying mechanisms remain poorly understood. This study aimed to compare LUTS in women with surgically confirmed endometriosis and symptomatic women in whom endometriosis was surgically excluded, and to investigate their association with urethral ultrasonographic parameters and lesion localization. **Methods:** This prospective case–control study included 163 women: 81 with surgically confirmed endometriosis and 82 symptomatic controls in whom endometriosis was surgically excluded. Urinary symptoms were assessed using the validated Urogenital Distress Inventory Short Form (UDI-6). Urethral length and mobility were evaluated by transvaginal pelvic floor ultrasonography at rest, during pelvic floor muscle contraction, and during the Valsalva maneuver. Associations between urinary symptoms, ultrasonographic findings, lesion localization, and selected clinical variables were analyzed using appropriate non-parametric tests and multivariable linear regression. **Results:** Women with endometriosis demonstrated significantly higher total UDI-6 scores than symptomatic controls (*p* = 0.002), indicating a greater burden of lower urinary tract symptoms. Significant differences were observed only for pain/discomfort and post-void urine leakage, whereas the remaining UDI-6 domains did not differ between groups. Overall urethral ultrasonographic parameters were comparable between groups. However, among symptomatic women (UDI-6 ≥ 1), urethral mobility during the Valsalva maneuver was significantly lower in women with endometriosis than in controls (*p* = 0.041). Endometriosis localization was not associated with urinary symptom severity or urethral ultrasonographic findings. In multivariable analysis, previous cesarean section, but not endometriosis itself, was independently associated with higher UDI-6 scores. **Conclusions:** Women with surgically confirmed endometriosis experienced a greater burden of lower urinary tract symptoms than symptomatic women without endometriosis, particularly pain/discomfort and post-void urine leakage. These symptoms were not associated with lesion localization or major differences in urethral morphology, indicating that structural urethral abnormalities alone do not explain urinary symptom severity in this population. Routine assessment of urinary symptoms using validated questionnaires may facilitate comprehensive evaluation of women with endometriosis. Further prospective multicenter studies incorporating standardized pain assessment, urodynamic evaluation, and longitudinal follow-up are warranted to better elucidate the mechanisms underlying lower urinary tract symptoms in this population.

## 1. Introduction

Endometriosis is a chronic, estrogen-dependent inflammatory disease affecting approximately 10% of women of reproductive age. It is characterized by the presence of endometrial-like tissue outside the uterine cavity and is associated with dysmenorrhea, dyspareunia, chronic pelvic pain, infertility, and impaired quality of life [1,2,3,4].

Although endometriosis has traditionally been regarded as a gynecological disorder, increasing evidence indicates that it should be considered a chronic systemic disease. Women with endometriosis frequently present with extra-gynecological symptoms and comorbidities, including gastrointestinal disorders, migraine, depression, anxiety, and chronic pain syndromes [3,4,5,6,7,8,9].

Lower urinary tract symptoms (LUTS) are increasingly recognized in women with endometriosis, even in the absence of direct urinary tract involvement. Reported symptoms include urinary frequency, urgency, dysuria, bladder discomfort, incomplete bladder emptying, and urinary incontinence [10,11,12,13,14,15,16]. These symptoms may substantially contribute to disease burden and remain underrecognized in routine gynecological care [8,9,10,11].

The mechanisms underlying LUTS in women with endometriosis remain incompletely understood. In patients with urinary tract endometriosis, symptoms may result from direct involvement of the bladder or ureters. However, urinary complaints may also occur without urinary tract infiltration, suggesting that chronic pelvic pain, pelvic floor dysfunction, neuroinflammatory mechanisms, and cross-organ sensitization may contribute to symptom development [12,13,14,15,17,18,19,20,21]. The overlap between endometriosis and interstitial cystitis/bladder pain syndrome may further complicate symptom attribution [17].

Pelvic floor ultrasonography is a non-invasive method used to assess urethral anatomy and dynamic urethral mobility. Although this technique is widely used in urogynecological practice, data regarding urethral ultrasonographic parameters in women with endometriosis remain limited [22,23,24].

Therefore, the primary aim of the present study was to compare lower urinary tract symptoms between women with surgically confirmed endometriosis and symptomatic women in whom endometriosis had been excluded surgically. Secondary objectives included evaluation of urethral ultrasonographic parameters, assessment of the relationship between urinary symptoms and lesion localization, and identification of clinical factors independently associated with urinary symptom severity.

## 2. Materials and Methods

### 2.1. Study Design

This prospective case–control study was conducted at the Department of Reproductive Medicine and Gynecology, Gynecological and Obstetric Clinical Hospital, Poznan University of Medical Sciences, Poland. The study was approved by the Bioethics Committee of the Poznan University of Medical Sciences (approval No. 12/26), and all participants provided written informed consent before enrollment. The study was conducted in accordance with the Declaration of Helsinki and Good Clinical Practice guidelines.

### 2.2. Study Population

A total of 163 women aged 17–49 years were included in the analysis. The study group consisted of 81 women with surgically confirmed endometriosis, whereas the control group included 82 symptomatic women in whom endometriosis was excluded during surgery.

The control group did not represent healthy volunteers. All women presented with symptoms suggestive of endometriosis, including dysmenorrhea, chronic pelvic pain, dyspareunia, or infertility, and therefore underwent laparoscopic evaluation. Only women in whom endometriosis was excluded intraoperatively were included as controls.

### 2.3. Inclusion and Exclusion Criteria

Women were eligible for inclusion in the endometriosis group if they fulfilled all of the following criteria:

- age between 17 and 49 years;
- clinical suspicion of endometriosis requiring surgical treatment;
- surgically confirmed endometriosis.

Women with cesarean scar endometriosis were included if the diagnosis was confirmed histopathologically following surgical excision.

The control group consisted of women undergoing laparoscopy because of symptoms suggestive of endometriosis, in whom no endometriotic lesions were identified during surgery.

Patients with pregnancy, malignant disease, active urinary tract infection, previous anti-incontinence surgery, neurological disorders affecting lower urinary tract function, or incomplete clinical data were excluded.

### 2.4. Classification of Endometriosis

For subgroup analyses, endometriosis was classified according to the predominant anatomical localization of the disease into:

- peritoneal endometriosis (other pelvic locations),
- Retzius space endometriosis,
- adenomyosis,
- cesarean scar endometriosis.

Localization data were available for all women with surgically confirmed endometriosis.

### 2.5. Clinical Assessment

All participants underwent standardized clinical assessment performed before surgery.

The following variables were recorded:

- age,
- body weight,
- height,
- BMI,
- parity,
- previous vaginal delivery,
- previous cesarean delivery,
- previous miscarriage,
- urinary symptoms,
- gynecological symptoms.

Urinary symptoms were evaluated using the Urogenital Distress Inventory Short Form (UDI-6), a validated patient-reported outcome measure assessing lower urinary tract symptoms in women [25]. The UDI-6 consists of six items scored from 0 (“not at all”) to 3 (“greatly bothered”). The total score is transformed to a 0–100 scale according to the original scoring algorithm, with higher scores indicating greater symptom severity [25].

The total UDI-6 score and individual questionnaire items were analyzed separately. The validated Polish language version of the UDI-6 questionnaire was used in the present study.

### 2.6. Ultrasonographic Assessment

All participants underwent transvaginal ultrasonography before surgery.

Assessment included evaluation of:

- urethral length,
- urethral mobility at rest,
- urethral mobility during pelvic floor muscle contraction,
- urethral mobility during the Valsalva maneuver.

Dynamic urethral mobility was measured relative to the inferior margin of the pubic symphysis.

Negative values represented posterior urethral displacement during dynamic examination.

Pelvic floor ultrasound examinations (PFS-TV) were performed using introital ultrasound by one operator trained in pelvic floor sonography. All assessments were conducted according to a standardized pelvic floor ultrasonography protocol, with patients examined in the same position and using identical measurement landmarks [22,23,24]. Urethral position and mobility were assessed at rest, during voluntary pelvic floor muscle contraction, and during the Valsalva maneuver. Ultrasound examinations were performed using a Voluson S8 ultrasound system (GE HealthCare, Chicago, IL, USA).

### 2.7. Surgical Diagnosis

Endometriosis was confirmed laparoscopically in women with pelvic disease.

Cesarean scar endometriosis was diagnosed on the basis of clinical history, characteristic ultrasound findings, and histopathological confirmation following complete surgical excision.

### 2.8. Statistical Analysis

Statistical analyses were performed using SigmaPlot v11.0 (Systat Software Inc., San Jose, CA, USA). The distribution of continuous variables was assessed using the Shapiro–Wilk test. As most variables were not normally distributed, continuous data are presented as median and interquartile range (IQR).

Comparisons between two independent groups were performed using the Mann–Whitney U test. Comparisons among endometriosis localization subgroups were performed using the Kruskal–Wallis analysis of variance on ranks. Categorical variables were analyzed using Pearson’s χ^2^ test or Fisher’s exact test, as appropriate.

To identify clinical factors independently associated with the severity of lower urinary tract symptoms, a multivariable linear regression model was constructed with the total Urogenital Distress Inventory-6 (UDI-6) score as the dependent variable. Based on their clinical relevance and potential association with urinary symptoms, the following independent variables were included in the model: age, body mass index (BMI), endometriosis status, previous vaginal delivery, previous cesarean delivery, and urethral length.

All statistical tests were two-sided, and a *p* value < 0.05 was considered statistically significant.

## 3. Results

### 3.1. Study Population

A total of 163 women were included in the final analysis, comprising 81 women with surgically confirmed endometriosis and 82 symptomatic controls in whom endometriosis was excluded surgically.

The endometriosis cohort was predominantly composed of women with stage I disease according to the revised American Society for Reproductive Medicine (rASRM) classification.

Within the endometriosis group, lesion localization included peritoneal endometriosis at sites other than the Retzius space, Retzius space endometriosis, adenomyosis, and cesarean scar endometriosis. Localization data were available for all patients.

Women with endometriosis were significantly younger than controls (median 30.0 vs. 32.0 years; *p* = 0.013). Body weight and BMI did not differ significantly between groups (*p* = 0.058 and *p* = 0.096, respectively). Nulliparity was more common among women with endometriosis, whereas multiparity was more frequent in controls. No significant differences were observed regarding previous cesarean delivery, vaginal birth, or miscarriage history (Table 1).

### 3.2. Lower Urinary Tract Symptoms

The results of the Urogenital Distress Inventory-6 (UDI-6) are presented in Table 2. Women with surgically confirmed endometriosis demonstrated significantly higher overall UDI-6 scores than symptomatic controls (*p* = 0.002), indicating a greater burden of lower urinary tract symptoms.

Analysis of individual questionnaire items revealed statistically significant differences only for small amounts of urine leakage (post-void dribbling) (*p* < 0.001) and pain or discomfort in the lower abdominal or genital area (*p* < 0.001). No significant between-group differences were observed for urinary frequency, urgency-associated urinary leakage, stress urinary incontinence, or difficulty emptying the bladder.

Sensitivity analysis excluding women with cesarean scar endometriosis demonstrated that the difference in total UDI-6 score between women with endometriosis and symptomatic controls remained statistically significant (Supplementary analysis, *p* = 0.001).

### 3.3. Ultrasonographic Findings

Ultrasonographic measurements are summarized in Table 3. No statistically significant differences were observed between women with endometriosis and symptomatic controls with regard to urethral length (*p* = 0.832), urethral mobility during pelvic floor muscle contraction (*p* = 0.254), or urethral mobility during the Valsalva maneuver (*p* = 0.321).

### 3.4. Multivariable Analysis

A multivariable linear regression model was constructed to identify independent factors associated with lower urinary tract symptom severity, expressed as the total UDI-6 score (Table 4). Previous cesarean section was the only independent predictor of higher UDI-6 scores (β = 24.85, 95% CI 4.69–45.02; *p* = 0.017). Neither age, body mass index, nor endometriosis status remained independently associated with urinary symptom severity. The overall regression model reached statistical significance (*p* = 0.049), explaining approximately 17% of the variability in total UDI-6 scores (R^2^ = 0.168).

### 3.5. Association Between Endometriosis Localization and Urinary Findings

Endometriosis localization was not significantly associated with the severity of lower urinary tract symptoms or urethral ultrasonographic parameters (Table 5). No statistically significant differences were observed between women with peritoneal endometriosis, Retzius space endometriosis, adenomyosis, or cesarean scar endometriosis regarding total UDI-6 score, urethral length, or urethral mobility during pelvic floor muscle contraction.

Although urethral mobility during the Valsalva maneuver tended to differ between localization subgroups, the difference did not reach statistical significance (*p* = 0.058).

### 3.6. Analysis of Symptomatic Women

A subgroup analysis was performed in women reporting at least one lower urinary tract symptom (UDI-6 score ≥ 1), including 71 women with endometriosis and 71 symptomatic controls.

No significant differences were observed between groups regarding urethral length at rest, during pelvic floor muscle contraction, or during the Valsalva maneuver (Table 6). Similarly, urethral mobility during pelvic floor muscle contraction did not differ significantly between groups.

In contrast, women with endometriosis demonstrated significantly lower urethral mobility during the Valsalva maneuver than symptomatic controls (5.0 [3.0–8.0] vs. 7.0 [4.0–10.0] mm, *p* = 0.041).

## 4. Discussion

The present study evaluated lower urinary tract symptoms (LUTS) and urethral ultrasonographic findings in women with surgically confirmed endometriosis. The principal finding was that women with endometriosis reported a significantly greater burden of lower urinary tract symptoms than symptomatic controls, as reflected by higher total UDI-6 scores. However, this difference was primarily driven by symptoms related to pain/discomfort and post-void urine leakage rather than by symptoms typically associated with overactive bladder. Furthermore, no significant differences were observed in urethral anatomical parameters between women with and without endometriosis.

These findings are consistent with previous reports demonstrating that women with endometriosis more frequently experience lower urinary tract symptoms even in the absence of direct urinary tract involvement. Gabriel et al. reported higher rates of urinary urgency, dysuria, incomplete bladder emptying, and bladder discomfort in women with endometriosis, whereas stress urinary incontinence was not significantly associated with the disease [10]. Similar observations have been described in women with urinary tract endometriosis and deep infiltrating endometriosis, suggesting that urinary complaints represent an important component of the clinical spectrum of endometriosis rather than being exclusively related to direct bladder infiltration [11,12,13,14,15].

The absence of significant differences in urethral length and urethral mobility suggests that the increased burden of urinary symptoms in women with endometriosis cannot be explained by major structural abnormalities of the urethra alone [22,23,24]. This supports the concept that LUTS in this population may be related to functional mechanisms rather than measurable anatomical changes detectable by ultrasonography [12,13,14,15,17,18,19,20,21].

One possible explanation is pelvic floor dysfunction associated with chronic pelvic pain. Women with endometriosis may demonstrate increased pelvic floor muscle tone, myofascial dysfunction, and altered neuromuscular activity, which may contribute to urinary urgency, bladder discomfort, voiding dysfunction, and pain [20,21]. In addition, chronic inflammation, altered pain processing, pelvic nerve involvement, and cross-organ sensitization between pelvic organs may contribute to urinary symptoms in women with endometriosis [17,18,19]. However, these mechanisms were not directly assessed in the present study and should therefore be interpreted as biologically plausible explanations rather than study conclusions.

Another clinically relevant finding was the lack of association between anatomical localization of endometriotic lesions and urinary symptom severity. Women with Retzius space endometriosis, adenomyosis, cesarean scar endometriosis, and peritoneal endometriosis at other locations demonstrated comparable UDI-6 scores and urethral ultrasonographic findings. Although urethral mobility during the Valsalva maneuver approached statistical significance across localization subgroups, this finding should be interpreted cautiously because of the limited number of patients in individual subgroups.

Multivariable regression analysis showed that previous cesarean section was the only independent predictor of higher UDI-6 scores, whereas endometriosis itself was not independently associated with urinary symptom severity. This finding suggests that LUTS are likely influenced by several clinical factors rather than by endometriosis alone. Previous studies have shown that mode of delivery and pelvic adhesions may affect pelvic floor function and urinary symptoms, which may partly explain this observation [26,27].

From a clinical perspective, these findings support routine assessment of urinary symptoms in women with endometriosis. The use of validated questionnaires such as the UDI-6 may help identify patients requiring further urogynecological evaluation [25]. Urinary symptoms may also adversely affect quality of life and sexual activity, further supporting their systematic assessment [28]. At the same time, the absence of significant differences in urethral ultrasonographic parameters suggests that pelvic floor ultrasound should be interpreted within the broader clinical context rather than used as a standalone explanation for urinary symptom severity [22,23,24].

The strengths of the present study include its prospective design, inclusion of women with surgically confirmed endometriosis and surgically verified symptomatic controls, comprehensive assessment of lower urinary tract symptoms using a validated questionnaire, and standardized ultrasonographic evaluation of urethral morphology and mobility. To our knowledge, this is one of the few prospective studies combining patient-reported urinary symptoms with objective transvaginal ultrasonographic assessment of the urethra in women with surgically confirmed endometriosis.

Several limitations should be acknowledged. First, this was a single-center study conducted at a tertiary referral institution, which may limit the generalizability of the findings. Second, the relatively small sample size reduced the statistical power of subgroup analyses according to endometriosis localization, particularly for women with adenomyosis and cesarean scar endometriosis. Third, urodynamic testing and bladder diary assessment were not performed, precluding objective evaluation of bladder function. Fourth, information regarding pain severity and hormonal treatment for all participants was unavailable and therefore could not be incorporated into the multivariable analyses. Another limitation of the study is that the majority of women with endometriosis had stage I disease according to the revised ASRM classification. Therefore, our findings may primarily reflect women with early-stage disease and may not be generalizable to patients with more advanced endometriosis. Finally, the control group consisted of symptomatic rather than healthy women, which may have reduced the between-group differences. In addition, no formal adjustment for multiple comparisons was performed; therefore, the results of secondary and exploratory analyses should be interpreted with caution.

## 5. Conclusions

Women with surgically confirmed endometriosis demonstrated a greater burden of lower urinary tract symptoms than symptomatic women without endometriosis, particularly with regard to pain/discomfort and post-void urine leakage. These symptoms were not accompanied by significant differences in urethral morphology or lesion localization, suggesting that structural urethral abnormalities alone do not explain urinary symptom severity in this population. Although endometriosis was associated with higher unadjusted UDI-6 scores, it did not remain an independent predictor after adjustment for potential confounding factors, whereas previous cesarean section emerged as the only independent predictor of urinary symptom severity. Routine assessment of lower urinary tract symptoms using validated questionnaires should therefore be considered as part of the multidisciplinary evaluation of women with endometriosis. Further prospective multicenter studies incorporating standardized pain assessment, urodynamic evaluation, and longitudinal follow-up are warranted to better elucidate the mechanisms underlying urinary symptoms in this population.

## Figures and Tables

**Table 1:** Baseline characteristics of the study population.

Variable	Endometriosis Group	Symptomatic Controls	*p* Value
Age, years	30.0 (24.0–35.0)	32.0 (29.0–38.8)	0.013
Body weight, kg	61.0 (55.5–69.5)	65.5 (57.0–77.5)	0.058
Height, cm	165.0 (163.0–170.5)	167.5 (163.0–170.0)	0.540
BMI, kg/m^2^	22.2 (20.9–25.4)	23.0 (21.1–28.1)	0.096
Nulliparous, n (%)	58/81 (71.6%)	43/78 (55.1%)	0.034
Parous, n (%)	23/81 (28.4%)	35/78 (44.9%)	0.034
Previous vaginal delivery, n (%)	19/81 (23.5%)	31/78 (39.7%)	0.040
Previous cesarean section, n (%)	4/81 (4.9%)	7/78 (9.0%)	0.363
Previous miscarriage, n (%)	8/81 (9.9%)	9/78 (11.5%)	0.801

**Table 2:** Lower urinary tract symptoms assessed using the Urogenital Distress Inventory-6 (UDI-6).

UDI-6 Item	Endometriosis Group (*n* = 81)	Control Group (*n* = 82)	*p*-Value
Total UDI-6 score	27.8 (11.1–33.3)	16.7 (5.6–22.2)	0.002
Frequent urination	1.0 (0.0–2.0)	1.0 (0.0–2.0)	0.861
Urine leakage associated with urgency	0.0 (0.0–1.0)	0.0 (0.0–0.0)	0.114
Urine leakage related to physical activity, coughing or sneezing	0.0 (0.0–1.0)	0.0 (0.0–1.0)	0.943
Small amounts of urine leakage (post-void dribbling)	0.0 (0.0–1.0)	0.0 (0.0–0.0)	<0.001
Difficulty emptying the bladder	0.0 (0.0–1.0)	0.0 (0.0–1.0)	0.239
Pain or discomfort in the lower abdominal or genital area	1.0 (0.0–2.0)	0.0 (0.0–1.0)	<0.001

Abbreviations: UDI-6, Urogenital Distress Inventory Short Form. Data are presented as median (interquartile range). Comparisons between groups were performed using the Mann–Whitney U test.

**Table 3:** Urethral ultrasonographic parameters in women with surgically confirmed endometriosis and symptomatic controls.

Variable	Endometriosis Group (*n* = 81)	Control Group (*n* = 82)	*p*-Value
Urethral length (mm)	34.0 (30.8–36.0)	33.5 (29.0–36.0)	0.832
Urethral mobility during pelvic floor muscle contraction (mm)	2.0 (−5.0 to 11.0)	2.0 (−15.0 to 14.0)	0.254
Urethral mobility during the Valsalva maneuver (mm)	−8.0 (−54.0 to 4.0)	−9.0 (−35.0 to 7.0)	0.321

Data are presented as median (interquartile range). Comparisons between groups were performed using the Mann–Whitney U test. Negative values indicate posterior urethral displacement relative to the inferior margin of the pubic symphysis during dynamic examination.

**Table 4:** Multivariable linear regression analysis of factors associated with total Urogenital Distress Inventory-6 (UDI-6) score.

Variable	β Coefficient	95% CI	*p*-Value
Previous cesarean section	24.85	4.69 to 45.02	0.017
Age (years)	0.38	−0.37 to 1.14	0.310
BMI (kg/m^2^)	−0.22	−1.32 to 0.88	0.690
Endometriosis	−7.08	−18.67 to 4.51	0.226

**Table 5:** Association between anatomical localization of endometriosis and lower urinary tract symptoms and ultrasonographic parameters.

Variable	Peritoneal Endometriosis (*n* = 40)	Retzius Space (*n* = 18)	Adenomyosis (*n* = 6)	Cesarean Scar (*n* = 8)	*p* Value
Total UDI-6 score	2 (0–3)	1 (0–3)	2 (0–3)	1 (0–3)	0.399
Urethral length (mm)	34 (20–41)	32 (27–41)	37 (25–39)	31 (27–38)	0.322
Urethral mobility during contraction (mm)	2 (−5 to 10)	2 (−5 to 11)	1 (0–3)	2 (−2 to 6)	0.779
Urethral mobility during the Valsalva maneuver (mm)	−8 (−58 to 4)	−11 (−58 to −4)	−5 (−14 to −3)	−11 (−18 to −6)	0.058

Data are presented as median (range). Comparisons were performed using the Kruskal–Wallis analysis of variance on ranks.

**Table 6:** Ultrasonographic urethral parameters in symptomatic women.

Parameter	Endometriosis (*n* = 71)	Control Group (*n* = 71)	*p* Value
Urethral length at rest (mm)	27.0 (24.0–30.0)	27.0 (25.0–30.0)	0.701
Urethral length during contraction (mm)	26.0 (23.0–29.0)	27.0 (24.0–30.0)	0.446
Urethral length during the Valsalva maneuver (mm)	25.0 (22.0–28.0)	26.0 (23.0–29.0)	0.391
Urethral mobility during contraction (mm)	2.0 (1.0–4.0)	3.0 (1.0–5.0)	0.172
Urethral mobility during the Valsalva maneuver (mm)	5.0 (3.0–8.0)	7.0 (4.0–10.0)	0.041

Data are presented as median (interquartile range). Comparisons were performed using the Mann–Whitney U test.

## Data Availability

The data presented in this study are available from the corresponding author upon reasonable request.
